# Can older patients adopt and maintain a ketogenic diet? An observational study in support of clinical trials in older patients

**DOI:** 10.1097/MD.0000000000028033

**Published:** 2021-11-24

**Authors:** Yahya Almodallal, Kathryn Cook, Lisa M. Lammert, Minji Lee, Jennifer G. Le-Rademacher, Aminah Jatoi

**Affiliations:** a Department of Oncology, Mayo Clinic, Rochester, MN; b Diet and Clinical Nutrition Unit, Mayo Clinic, Rochester, MN; c Department of Health Sciences Research, Mayo Clinic, Rochester, MN.

**Keywords:** cancer, diabetes, diet, geriatric, ketogenic, obesity, older

## Abstract

Ketogenic diets appear promising for obesity, diabetes, cancer, and other illnesses. Because older patients are more likely to contend with such illnesses and because of a paucity of dietary outcomes among these patients, we examined ketogenic diets in older patients.

This multisite study focused on patients (≥65 years of age) on a ketogenic diet. Medical records were identified with the keywords “keto,” “ketogenic,” and “Atkins.” Records were reviewed in detail with extraction of direct quotations to substantiate observations.

We report on 200 consecutive patients with a median age of 70 years. Reasons for diet included weight loss, diabetes, and cancer; the majority remained on the diet for >1 month. In 134 (67%: 95% confidence interval: 60, 73%), the ketogenic diet appeared beneficial: 93 of 117 (79%) who sought weight loss lost weight (“She has lost 15 pounds and plans to lose another 8”); 36 of 67 (54%) who sought glucose control appeared to achieve the latter (“He has gone on a ketogenic diet and has been able to bring his sugars down significantly”); and 5 of 8 (63%) who sought improved cancer outcomes appeared to derive them (“He attributes part of the control of his cancer and increased QOL to adopting the keto for cancer diet”). Adverse events occurred in 30 patients (15%): dyslipidemia (n = 14), constipation (n = 9), sub-therapeutic international normalized ratio (n = 3), pancreatitis (n = 2), diarrhea (n = 1), and fatigue (n = 1).

Trials that test ketogenic diets for a variety of illnesses should enroll older adults.

## 1 Introduction

Previous studies suggest older patients are slow to complete unfamiliar tasks, raising concerns that health-promoting recommendations aimed at changing life-long habits might be challenging to implement.^[1]^ Still other studies draw the opposite conclusion: older patients, particularly those who are not cognitively compromised, appear to be more receptive and adherent to lifestyle modifications than their younger counterparts.^[2]^

This question of adherence to lifestyle changes is especially relevant to the ketogenic diet, which has evolved over time in large part because of adherence issues.^[3,4]^ The classical ketogenic diet was developed in the 1920s primarily for the treatment of epilepsy and called for the initiation of the diet as an inpatient, a requirement to weigh food portions, and strict restrictions on fluid and caloric consumption. The goal was to have patients ingest a 4:1 ratio of fat to protein plus carbohydrates. The burdensome nature of this diet, coupled with a growing interest to expand its indications, prompted the development of a modified ketogenic diet – otherwise known as the modified Atkins diet – which did not require hospitalization, did not require the weighing of food, and provided no fluid or calorie restrictions. The above macronutrient ratio was also liberalized to 1:1. These changes resulted in a diet that was easier for patients but nonetheless designed to meet the intended therapeutic goals of the diet.

Such therapeutic goals are perhaps best realized in the role of ketogenic diets in controlling seizures in children, although mounting evidence suggests that adults might also derive such therapeutic benefit.^[5–10]^ In adults, a ketogenic diet appears to palliate not only seizures but also obesity, diabetes, cognitive dysfunction, and even cancer.^[5–10]^ Despite such promise and despite the fact that many of the illnesses above occur predominantly in older patients, few studies have examined this diet in older adults.^[10,11]^ This paucity of information underscores the need to begin to examine whether older patients can follow such a diet. Thus, the current study was undertaken to seek justification for designing clinical trials with this diet in older patients.^[12]^

## 2 Methods

### 2.1 Overview

This multisite study from the Mayo Clinic took advantage of this medical center's broad catchment area that serves patients in Minnesota, Florida, Arizona, surrounding states, and beyond. This study was intended to explore whether older patients (defined as ≥65 years of age based on Medicare criteria) appear capable of adopting and maintaining a ketogenic diet and whether such a diet appears to show evidence of therapeutic benefit as well as safety. A review of patients’ medical records was undertaken for this purpose. The Mayo Clinic Institutional Review Board provided approval to proceed with the study (#20-010021).

### 2.2 Medical record ascertainment and data acquisition

Patient eligibility criteria consisted of the following: patient age of 65 years or older at the time of most recent mention of the diet in the medical record; inclusion of the terms “keto,” “ketogenic,” or “Atkins” in the medical record; and medical record evidence that the patient had initiated such a diet.

The review of medical records was undertaken as a twofold process. First, the first 2 eligibility criteria were addressed with the use of the appropriate medical record search criteria, as applied to thousands of medical records. We accessed medical records from March 1, 2020 and then reviewed consecutive, older records to reach our intended sample size. A single investigator (KC) then screened each of the resulting consecutive medical records by hand to select those patients who met the third criterion. This approach enabled us to acquire long-term outcome and adverse event data on patients who might have started the ketogenic diet when younger but had been on the diet for many months or even years with continued adherence to the age of 65 or later.

Second, another investigator (YA) performed an in depth review by hand of each medical record to confirm patient eligibility and then to extract detailed demographic information, the underlying indication for adopting the diet, patient morbidity, whether a dietician had provided dietary instruction on the ketogenic diet, diet duration, whether the diet appeared to achieve its intended goal, and adverse events associated with the diet. Overlapping indications for the diet prompted the study team to use clinical discretion to cite the predominant indication. Diet duration was based on dates in the medical record as well as patient reports of time on the diet, as gleaned from the medical record. A third investigator (AJ) performed discretionary checks of data elements to ensure accuracy of data capture.

### 2.3 Sample size and analyses

Sample size was based on the objective to estimate the rate of benefit as broadly defined based on indication for the diet, that is, the proportion of patients in whom the ketogenic diet appeared to accomplish its intended goal, with a half-width of a 95% confidence interval within 10%. This half-width was chosen based on what appeared to be clinically relevant in the opinion of the study team. With a sample size of 200 patients, the upper and lower boundaries of a confidence interval fall within no more than 8% under the most conservative scenario of an assumed rate of benefit of 50%. Data are presented descriptively with medians, ranges, percentages, and 95% confidence intervals, as appropriate. Direct quotations from the medical record were used to aid in the assessment of diet palliation for a given medical indication and are presented in a verbatim, representative manner.

## 3 Results

### 3.1 Demographics

**Figure 1:**
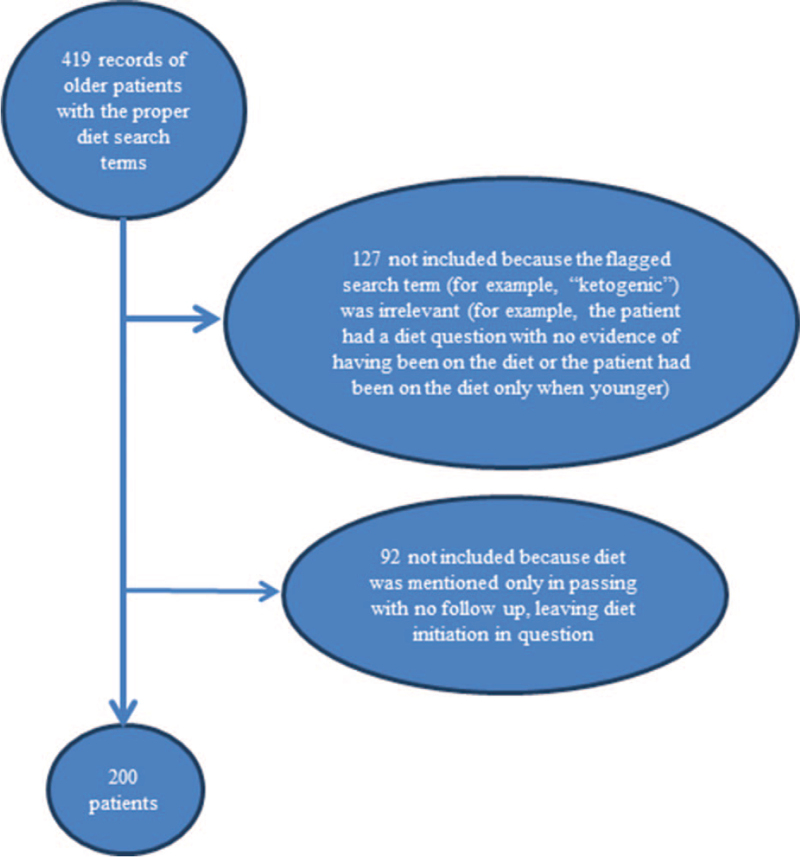
Consort diagram.

The medical records of 200 consecutive patients, who were on a version of a ketogenic diet, from November 7, 2019 to March 1, 2020, are the subject of this report (Fig. 1). The median patient age at diet initiation was 70 years (range: 61, 90 years); only 2 patients were younger than 65 years of age (Table 1).

**Table 1:** Demographics (n = 200)^∗^.

Median age (range)^†^	70 (61, 90)
Sex	
Male	98 (49)
Female	102 (51)
Indication for ketogenic diet	
Weight loss	117 (59)
Diabetes control	67 (34)
Cancer treatment	8 (4)
Other^‡^	8 (4)
Median number of morbidities (range)^§^	4 (1,11)

∗ Numbers in parentheses denote percentages unless otherwise noted.

† In year, at first mention of ketogenic diet in the medical record with only 2 patients younger than 65 years of age.

‡ Other includes cognition (n = 1), arthritis (n = 2), and unclear reasons (n = 5).

§ Includes hypertension in 138 patients (69%), dyslipidemia in 125 (63%), and heart disease in 67 (34%).

Reasons for adopting a ketogenic diet consisted of the following: a need/desire for weight loss in 117 patients (59%), diabetes management in 67 (34%), cancer treatment in 8 (4%), and other indications in 8 (4%) (Table 1). Patients had a median number of morbidities of 4 (range: 1, 11) inclusive of the primary reason for diet adoption (Table 1). Of note, 125 patients (63%) had dyslipidemia.

### 3.2 Ketogenic diet details

Only 21 patients (11%) underwent formal dietary education for a ketogenic diet, most commonly with a dietician. Specifics on actual dietary intake were virtually absent in the medical record. At the time of this report, 75 patients (38%) had been on a ketogenic diet for greater than a month but less than a year, and 44 (22%) had been on it for a year or longer (Table 2). In 74 patients (37%), duration was not possible to discern.

**Table 2:** Ketogenic diet information^∗^.

Did a healthcare provider provide education on the ketogenic diet?	
Yes	21 (11)
No/unknown	179 (89)
Duration of diet	
<1 week	0
1 week to 1 month	7 (4)
>1 month but less than a year	75 (38)
>1 year or longer	44 (22)
Cannot tell	74 (37)
Complications from the diet^†^	
Dyslipidemia	14 (7)
Constipation	9 (5)
Low international normalized ratio	3 (2)
Pancreatitis	2 (1)
Diarrhea	1
Fatigue	1

∗ Numbers in parentheses denote percentages unless otherwise noted.

† Numbers denote per patient adverse events.

### 3.3 Therapeutic outcomes with a ketogenic diet

In 134 patients (67%: 95% confidence interval: 60, 73%) the ketogenic diet appeared to accomplish its intended goal. Favorable outcomes appeared in 93 of the 117 patients (79%) who sought weight loss, 36 of 67 diabetes patients (54%) who sought better glucose control, and 5 of 8 patients (63%) who sought antineoplastic effects (Table 3).

**Table 3:** Therapeutic dietary outcomes for obesity/overweight, diabetes, and cancer.

Outcome	Number of patients who achieved goal (%)^∗^	Representative verbatim quotations from the medical record^†^
Weight loss	93 (79)	“She has lost about 15 pounds and plans to lose another 8.” “She states she has been able to lose several pounds on the keto diet.” “He started doing a keto diet and has lost 4 kilos.” “She has also been on a keto diet and has been successful in significant weight loss and feels great.” “She is on the Atkins diet. Her weight has come down.” “She reports she is actually down 20 pounds on her scale at home.” “She has lost 35 lb since last April and this weight loss has improved her joint pain. Weight loss was achieved through the keto diet.” “He has intentionally lost approximately 20 lb… he is following the ‘keto diet.’ Positive for increased energy.” “She went on a keto diet which improved her arthritis and lost several pounds.” “He managed to successfully tackle this with a ketogenic diet and has actually lost 35 lb already.”
Glucose control	36 (54)	“Her blood glucose levels are improved.” “Her primary doctor took her off her meds because of improvement of her blood sugars because of her diet.” “Her glucose numbers have improved significantly. Checking twice a day her average is 141.” “Since receiving news of his elevated A1c, he has been eating a keto diet….he is not on any medications for diabetes.” “Blood glucose 130's.” “Patient saw improvement in his HDL, cholesterol, and A1C (was prediabetic) after just 2 weeks of following the keto diet.” “He has gone on a “ketogenic” diet and has been able to bring his sugars down significantly… He did have labs done recently that showed his A1c with excellent control at 6.1.” “The patient's A1c has increased slightly but is still showing great control at 5.9%. The patient tells me he continues to have a keto diet.” “Ketogenic diet since April….A1c drawn on 11/6 within normal limits.” “He is currently following the keto diet and feels this is helping keep his blood sugars and weight stable.” “She started a keto diet on January 1…when she checked last week [her blood glucose] was 120.” “Improving diabetes since on keto diet.” “Her A1C has actually improved as well.” “A1c indicates controlled diabetes.” “She is diabetic. Her hemoglobin A1c is 6.1. That is reviewed. She is not testing. She is on no medications… She is following a keto-type diet.” “I did review his log book with him. His blood sugars have decreased significantly recently…. He is on a keto diet.” “He has started following a keto diet with his wife…. He notes fasting glucose levels of 90–120.” “His A1c has improved slightly to 5.9%.” “By starting a keto diet… her A1C has actually improved as well.”
Antineoplastic effects	5 (63)	“He attributes part of the control of his cancer and increased QOL to adopting the keto for cancer diet.” “He states he has been on a ketogenic diet… again, he is resistant to starting chemotherapy because he is asymptomatic of his disease.” “He reports he is going to attack his cancer in that direction and is on some diet changes, a ketogenic diet…. he overall has done very well.” “I did confirm that the patient did not start hormone therapy despite our previous discussion… the patient feels comfortable with further observation and pursuing a keto diet… PSA has risen very slowly and now is at a value of 2.3.”

∗ Denominator for the percentage is based in the number of patients who sought that that specific goal.

† Each quotation is from a unique patient record.

### 3.4 Adverse events

Thirty patients (15%) developed an adverse event deemed related to a ketogenic diet. These adverse events included dyslipidemia (n = 14), constipation (n = 9), sub-therapeutic international normalized ratio (n = 3), pancreatitis (n = 2), diarrhea (n = 1), and fatigue (n = 1).

## 4 Discussion

This study demonstrates that older patients who have extensive comorbidity as well as a health problem that might be palliated from a ketogenic diet can adopt such a diet and manifest salutary outcomes, such as weight loss, improved glucose control, and perhaps even some antineoplastic effects. In the current study, as many as 67% of older patients, who started a ketogenic diet, achieved a medical outcome in alignment with their therapeutic goal. Importantly, 15% of patients developed a diet-related adverse event –which underscores the need for caution when prescribing a ketogenic diet – but, in the context of dietary adherence, further substantiates our conclusion that older patients are capable of adopting and adhering to a ketogenic diet. This study outlines a role for including older patients in future prospective studies with ketogenic diets.

Indeed, the genesis of the study reported here was to address the unmet need for evidence that a ketogenic diet could be implemented in older patients. Particularly in a cancer setting, older patients contend with side effects from cancer treatment that could conceivably be favorably impacted by diet. For example, a new class of cancer agents, the PI3K inhibitors, causes high rates of hyperglycemia, particularly in older patients, leading at times to detrimental polypharmacy and even hospitalization.^[13]^ The findings from the current study suggest that some older patients are able to adopt a ketogenic diet. If tested within the context of a clinical trial, healthcare providers could readily learn whether such a diet-based intervention could prevent, attenuate, or reduce the morbidity of this drug-induced hyperglycemia.^[14]^ In short, this study provides evidence of feasibility that dietary modification – and specifically a ketogenic diet – merits further testing in clinical trials aimed at making cancer treatment as well as perhaps other health issues more tolerable for older patients.

Of note, the older patients in this study had notable baseline morbidity. For example, the majority had baseline dyslipidemia. Although a ketogenic diet has been cited as a cause of dyslipidemia and although we observed worsening lipid profiles in 14 patients, interestingly, the majority of these patients did not suffer worsening dyslipidemia with the ketogenic diet. Although future clinical trials with ketogenic diets will most assuredly incorporate close monitoring, one might conclude that a ketogenic diet appears reasonably well tolerated in older patients.

This study has limitations. First, our study design runs the risk of having selected patients who were either more likely to manifest benefit or more likely to manifest adverse events from a ketogenic diet. From a practical standpoint, it appears unlikely that patients who tried the diet and derived no benefit and no ill effects would mention their inconsequential adoption of a new diet during a clinic visit. Either the success of the diet or the need for medical help would result in such a recorded discussion of the diet during a medical visit. Despite such potential selection bias, the findings from this study nonetheless make clear that some older patients are clearly capable of safely adopting and maintaining a ketogenic diet. Second, our reliance on medical record review poses another limitation. We do not know how these diets were taught to patients, whether the diet had been formally prescribed, or whether patients had relied on other sources of information to learn how to adhere to a ketogenic diet. It would be important to know whether patients relied on, for example, the internet, television, or magazines to learn about the diet as well as the content of the relied upon information from these non-medical sources. Future prospective studies should outline dietary instruction in a detailed manner. Third, the design of the current study limited our ability to discern exactly what type of ketogenic diet a patient had implemented. In view of previous research that shows the classical ketogenic diet is highly restrictive and therefore challenging to accept and in view of the fact that high-profile individuals’ have touted a version of the modified ketogenic diet (also known as the modified Atkins diet), it appears likely that patients had adopted some version of the latter. Along similar lines, a better understanding of adherence to the ketogenic diet would be of value, as the current study was unable to acquire such information. In future prospective studies, investigators should consider providing specific information about the type of ketogenic diet prescribed or the type of diet patients had chosen to adopt and how well that diet was adhered to, acknowledging that more recent renditions of the ketogenic diet have vastly improved patient adherence. Finally, although the current study suggests that the ketogenic diet resulted in the observed improvements in weight loss and glucose control, we were unable to establish cause and effect. It could be that the observed improvements occurred as a result of other concomitant interventions, such as the initiation of an exercise program or other dietary modifications. Again, a prospective study could be key to better understanding the observed findings of the current study.

In summary, this study demonstrates that some older patients are capable of initiating and maintaining a ketogenic diet. Although older patients appear to have gleaned benefits from a ketogenic diet, this study was not intended to generate recommendations to prescribe or to not prescribe such a diet for medical purposes. Rather, the goal was to provide the rationale to justify further exploration of dietary adherence in a prospective manner with the long term objective of providing older patients opportunities to enroll in clinical trials that test a ketogenic diet. Our findings suggest a need to further study ketogenic diets – and most specifically adherence, benefits, and risks – in older adults.

## Author contributions

**Conceptualization:** Yahya Almodallal, Jennifer G. Le-Rademacher, Aminah Jatoi.

**Data curation:** Yahya Almodallal, Kathryn Cook, Lisa M. Lammert, Minji Lee, Jennifer G. Le-Rademacher, Aminah Jatoi.

**Formal analysis:** Yahya Almodallal, Kathryn Cook, Lisa M. Lammert, Minji Lee, Jennifer G. Le-Rademacher, Aminah Jatoi.

**Funding acquisition:** Aminah Jatoi.

**Investigation:** Yahya Almodallal, Kathryn Cook, Lisa M. Lammert, Minji Lee, Jennifer G. Le-Rademacher, Aminah Jatoi.

**Methodology:** Yahya Almodallal, Kathryn Cook, Minji Lee, Aminah Jatoi.

**Project administration:** Yahya Almodallal, Kathryn Cook, Aminah Jatoi.

**Resources:** Yahya Almodallal, Kathryn Cook, Lisa M. Lammert, Aminah Jatoi.

**Supervision:** Aminah Jatoi.

**Validation:** Yahya Almodallal, Lisa M. Lammert, Minji Lee, Jennifer G. Le-Rademacher, Aminah Jatoi.

**Visualization:** Yahya Almodallal, Aminah Jatoi.

**Writing – original draft:** Yahya Almodallal, Aminah Jatoi.

**Writing – review & editing:** Yahya Almodallal, Kathryn Cook, Lisa M. Lammert, Minji Lee, Jennifer G. Le-Rademacher, Aminah Jatoi.

## References

[1] Wessel JR Dolan KA Hollingworth A. A blunted phasic autonomic response to errors indexes age-related deficits in error awareness. Neurobiology of Aging 2018;71:130–20. 10.1016/j.neurobiolaging.2018.06.019 30071369

[2] Leung AWY Chan RSM Sea MMM Woo J. An overview of factors associated with adherence to lifestyle modification programs for weight management in adults. Int J Environ Res Public Health 2017;14:922. 10.3390/ijerph14080922 PMC5580624 28813030

[3] Batch JT Lamsal SP Adkins M Sultain S Ramirez MN. Advantages and disadvantages of the ketogenic diet: a review article. Cureus 2020;12:e9639. 32923239 10.7759/cureus.9639 PMC7480775

[4] McDonald RJW Henry-Barron BJ Felton EA. Improving compliance in adults with epilepsy on a modified Atkins diet: a randomized trial. Seizure 2018;60:132–8. 29960852 10.1016/j.seizure.2018.06.019

[5] Dashti HM Mathew TC Al-Zaid NS. Efficacy of low carbohydrate ketogenic diet in the treatment of type 2 diabetes. Med Princ Pract 2021;30:223–35. 33040057 10.1159/000512142 PMC8280429

[6] Casanueva FF Castellana M Bellido D. Ketogenic diets as treatment of obesity and type 2 diabetes mellitus. Rev Endocrin Metab Disord 2020;21:381–97. 10.1007/s11154-020-09580-7 32803691

[7] Dashti HM Mathew TC Hussein T. Long-term effects of a ketogenic diet in obese patients. Exp Clin Cardiol 2004;9:200–5. 19641727 PMC2716748

[8] Thomas JG Veznedaroglu E. Ketogenic diet for malignant gliomas: a review. Current Nutr Rep 2020;9:258–63. 10.1007/s13668-020-00332-2 32720120

[9] Morrison SA Fazeli PL Gower B. Cognitive effects of a ketogenic diet on neurocognitive impairment in adults aging with HIV: a pilot study. J Assoc Nurses AIDS Care 2020;31:312–24. 31725105 10.1097/JNC.0000000000000110 PMC7883774

[10] Zahra A Fath MA Opat E. Consuming a ketogenic diet while receiving radiation and chemotherapy for locally advanced lung cancer and pancreatic cancer: the University of Iowa experience of two phase 1 clinical trials. Radiat Res 2017;187:743–54. 28437190 10.1667/RR14668.1 PMC5510645

[11] Kverneland M Selmer KK Nakken KO Iversen PO Taubøll E. A prospective study of the modified Atkins diet for adults with idiopathic generalized epilepsy. Epilepsy Behav 2015;53:197–201. 26588588 10.1016/j.yebeh.2015.10.021

[12] Pasca L Varesio C Ferraris C. Families’ perception of classic ketogenic diet management of acute medical conditions: a web-based survey. Nutrients 2020;12:E2920. 32987704 10.3390/nu12102920 PMC7598657

[13] Andre F Ciruelos E Rubovszky G. Alpelisib for PIK3CA-mutated, hormone receptive-positive advanced breast cancer. N Engl J Med 2019;380:1929–40. 31091374 10.1056/NEJMoa1813904

[14] Hopkins BD Pauli C Du X. Suppression of insulin feedback enhances the efficacy of PI3K inhibitors. Nature 2018;560:499–503. 30051890 10.1038/s41586-018-0343-4 PMC6197057

